# Reducing Brain Signal Noise in the Prediction of Economic Choices: A Case Study in Neuroeconomics

**DOI:** 10.3389/fnins.2017.00704

**Published:** 2017-12-14

**Authors:** Raanju R. Sundararajan, Marco A. Palma, Mohsen Pourahmadi

**Affiliations:** ^1^Department of Statistics, Texas A&M University, College Station, TX, United States; ^2^Department of Agricultural Economics, Texas A&M University, College Station, TX, United States

**Keywords:** choice behavior, neuroeconomics, EEG data, multi-dimensional time series, stationary subspace analysis, C32, D87

## Abstract

In order to reduce the noise of brain signals, neuroeconomic experiments typically aggregate data from hundreds of trials collected from a few individuals. This contrasts with the principle of simple and controlled designs in experimental and behavioral economics. We use a frequency domain variant of the stationary subspace analysis (SSA) technique, denoted as DSSA, to filter out the noise (nonstationary sources) in EEG brain signals. The nonstationary sources in the brain signal are associated with variations in the mental state that are unrelated to the experimental task. DSSA is a powerful tool for reducing the number of trials needed from each participant in neuroeconomic experiments and also for improving the prediction performance of an economic choice task. For a single trial, when DSSA is used as a noise reduction technique, the prediction model in a food snack choice experiment has an increase in overall accuracy by around 10% and in sensitivity and specificity by around 20% and in AUC by around 30%, respectively.

## 1. Introduction

The interest of economists and other social scientists to integrate neurophysiological data to study human behavior has dramatically increased. Neuroeconomics has opened the door to research aiming to explain behavioral models of decision making (Camerer et al., 2004). However, neuroeconomics has only had limited reception into mainstream economics, perhaps due to the limitation of brain processes for improving the prediction of economic behavior (see Harrison, 2008; Bernheim, 2009; Konovalov and Krajbich, 2016). After all, economists are ultimately interested in predicting behavior (Gul and Pesendorfer, 2008; Fehr and Rangel, 2011).

Behavioral and experimental economics seem to be very much interrelated with neuroeconomics. However, behavioral and experimental economics rely on simple and controlled experiments to infer causality. Brain data, by nature, are very noisy. This is due to the fact that subjects react to the presented stimuli and process it in their brain visually (i.e., colors, shapes, etc.), physically (i.e., moving their eyes and muscles), emotionally, engaging in memory and other processes that simultaneously activate different regions of the brain. The data collected from an individual on a single trial measures the activity of the brain for the stimuli, but it also captures the noise from all other activity unrelated to the task of the experiment. Brain experiments typically implement hundreds of trials that when aggregated filter out noisy signals (Plassmann et al., 2007; Hare et al., 2009; Milosavljevic et al., 2010). There is a trade-off between the experimental economics principle of designing simple experiments to assess causality and the neuroeconomics need for a large number of trials to reduce the noise in brain signals.

An emerging literature in neuroeconomics uses brain signals to directly explain choice behavior. One of the models used to explain decision making is the Neural Random Utility Model (NRUM; Webb et al., 2013). EEG data have been used to predict purchase decisions (Ravaja et al., 2013), consumer's future choices (Telpaz et al., 2015), predict preferences (Khushaba et al., 2012, 2013) and response to advertisements (Boksem and Smidts, 2015; Venkatraman et al., 2015).

**Figure 1:**
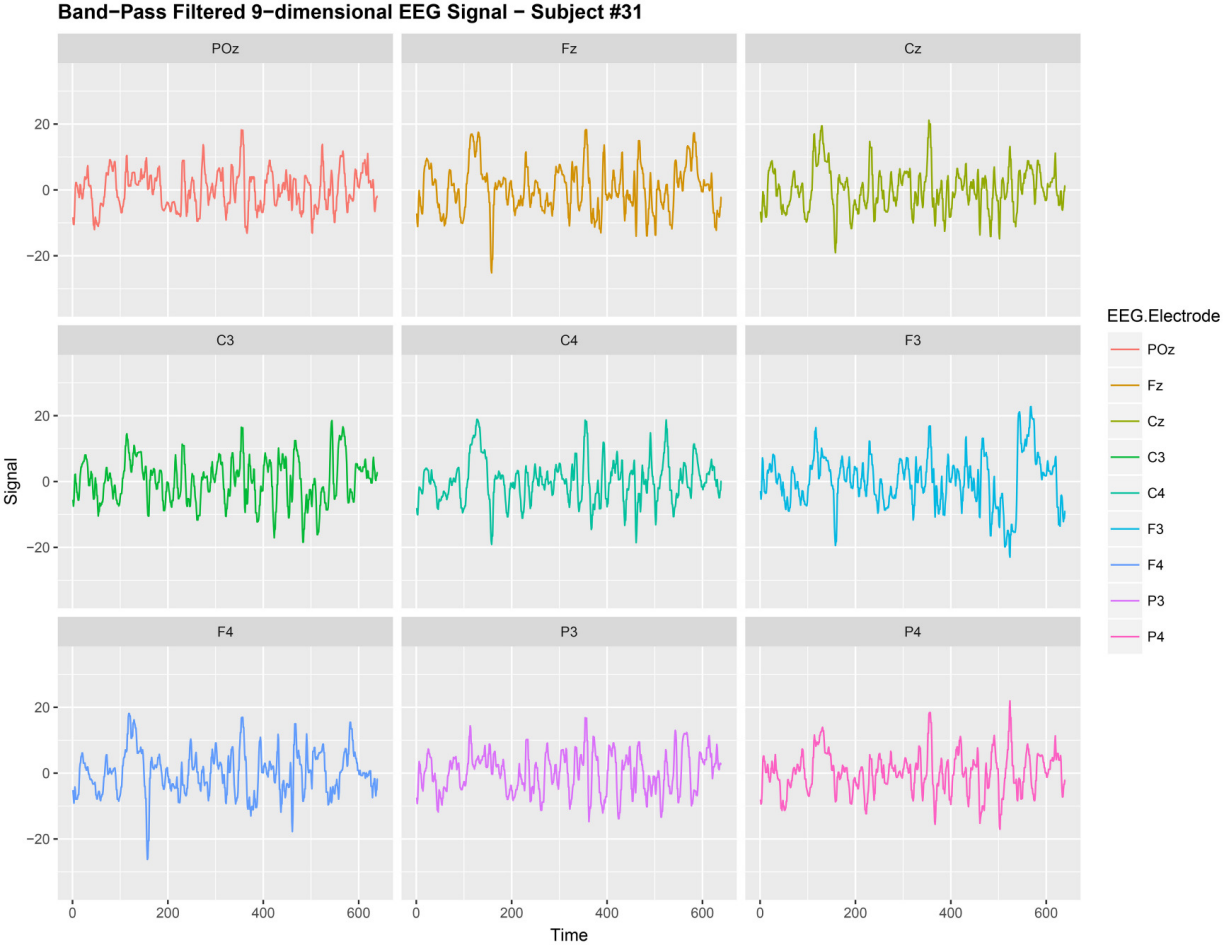
Band-Pass filtered 9-dimensional EEG signal {*X*_*t,j*_:*t* = 1, 2, …, 640} (before noise reduction) gathered from subject #31 while responsing to food-choice question number 9.

**Figure 2:**
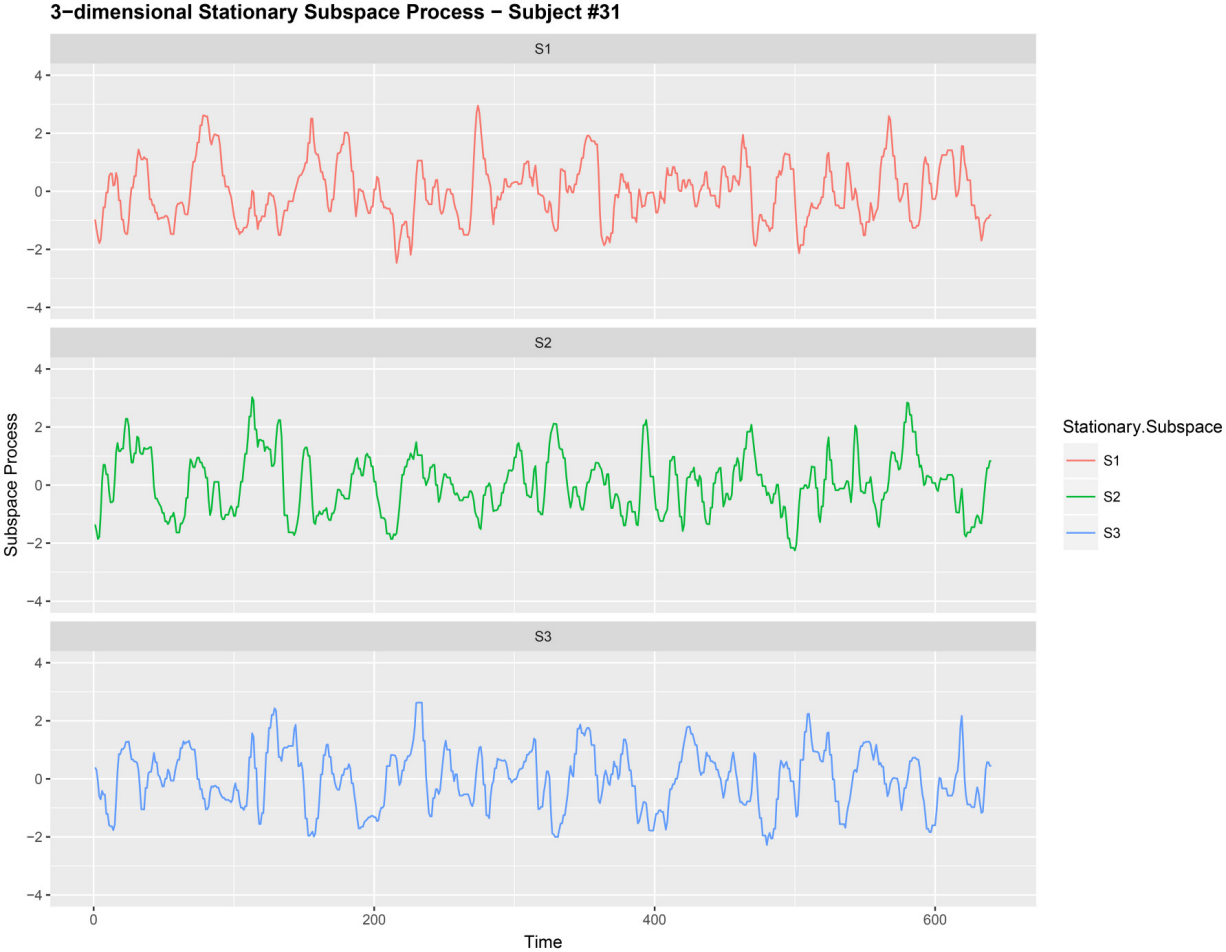
Three-dimensional stationary subspace process {*Y*_*t,j*_:*t* = 1, 2, …, 640} (after noise reduction) gathered from subject #31 while responsing to food-choice question number 9.

EEG signals from different electrodes measuring brain activity have, in the past, been regarded as a multi-dimensional nonstationary time series; see Ombao et al. (2005) and von Bünau et al. (2010) for examples. Kaplan et al. (2005) regard the nonstationarity as the “unavoidable noise” in the brain signal. Here the nonstationary sources in the brain signal contributes to the noise in the EEG data and removing this nonstationarity is extremely useful for prediction purposes in brain related experiments. We use the words noise and nonstationarity interchangeably because in our setup the nonstationary sources contribute to parts of the signal that are unrelated to the task related activity in the experiment. Hence eliminating nonstationarity reduces noise in the brain signal. See section 3.2 and Figures 1, 2 for illustrations of the signal before and after noise reduction. von Bünau et al. (2009) and von Bünau et al. (2010) associate *alpha* oscillations in the data as a nonstationary source. These oscillations appear usually in the range of 8–12 Hz and are associated with blinking, fatigue or tiredness. Over the course of the experiment such changes in the EEG time series are unrelated to the experimental task and corrupt the signal.

Transforming a multi-dimensional nonstationary time series into stationarity through linear transformations is a nontrivial problem of fundamental importance in many application areas. Stationary subspace analysis (SSA) (von Bünau et al., 2009) is a recent technique that attempts to find stationary linear transformations, in lower dimensions, of multi-dimensional nonstationary time series, where nonstationarity means independent heterogenous data with mean and variance smoothly varying across time.

In this paper, we apply dependent SSA (DSSA, for short) method proposed in Sundararajan and Pourahmadi (2017) for the general class of multi-dimensional nonstationary time series to data from a neuroeconomics case study. DSSA relies on the asymptotic uncorrelatedness of the discrete Fourier transform (DFT) of a second-order stationary time series at different Fourier frequencies and unlike the well-known *cointegration* theory that is restricted to parametric models such as VAR, DSSA avoids parametric model assumptions.

We employ the DSSA technique, as a noise reduction step to separate stationary (useful signal) and nonstationary sources to reduce noise in the EEG brain signal. This is important because using this process may move neurophysiological responses to become more aligned with the design of traditional economics experiments. In other words the nonstationary sources in the brain signal are associated with variations in the mental state that are unrelated to the experimental task at hand. Hence the DSSA technique can be useful in reducing the number of trials needed from each participant in neuroeconomic experiments. More importantly, the technique greatly improves the prediction performance of an incentivized economic food choice task. In addition, the DSSA technique performs a formal test of stationarity that ensures there is a statistically significant reduction in nonstationarity (noise) in the observed signal. The ability of DSSA and ISSA in detecting the true dimension of the stationary subspace process is illustrated for different sample sizes and dimensions and it was observed that in most cases DSSA performs better than ISSA. See section 3 of Sundararajan and Pourahmadi (2017) for more details.

The rest of the paper has the following content: Section 2 introduces the SSA model setup, describes the existing SSA technique and then the DSSA approach. Section 3 discusses an empirical application of decisions for purchasing food snacks. Subjects were presented with 10 food snack choice questions and the observed EEG signal from 9 electrodes is treated as a 9-dimensional time series. The various steps taken for noise reduction are described in section 3.2 and the “noise reduced” series is then analyzed. Finally, prediction models (section 3.2.2) of the decision made by the subjects regarding various food choices are constructed and their performance is illustrated.

## 2. Stationary subspace analysis (SSA)

We begin with the SSA model setup and the method in von Bünau et al. (2009) which deals with independent and heteroscedastic data and is denoted by ISSA. Then a SSA method for dependent data, denoted by DSSA, for finding the stationary subspace process (noise reduced signal) of the observed second-order nonstationary process (observed EEG signal) is described.

### 2.1. ISSA

Let {*X*_*t*_} be the observed p-dimensional nonstationary time series that is linearly generated by *d* stationary sources $Y_{t}^{s}$ and *p* − *d* nonstationary sources $Y_{t}^{n}$. More precisely,

(1) $$\begin{array}{r} X_{t}=AY_{t}=\left[\begin{array}{cc} A_{s} & A_{n} \end{array}\right]\left[\begin{array}{c} Y_{t}^{s}\\ Y_{t}^{n} \end{array}\right], \end{array}$$

where *A* is the unknown *p* × *p* (invertible) mixing matrix, *A*_*s*_ and *A*_*n*_ are *p* × *d* and *p* × (*p* − *d*) matrices, respectively. The dimension *d* is unknown and needs to be estimated. In ISSA, the notion of stationarity is with respect to the first two moments (i.e., mean and lag-0 covariance).

The goal is to estimate the demixing matrix *B* = *A*^−1^ so that *Y*_*t*_ = *BX*_*t*_ is separated into stationary and nonstationary sources. The nonstationary source $Y_{t}^{n}$ contributes to the noise (for example, fatigue and tiredness of participants during the experiment) in the observed EEG signal and separating it from the stationary source $Y_{t}^{s}$ is useful in improving performance in prediction models.

The matrix *B* is assumed to be an orthogonal matrix. The matrix *B* is estimated first by dividing the time series data into *N* epochs and then minimizing, as a function of *B*, the Kullback-Leibler (KL) divergence between Gaussian distributions across these segments. Let ${\hat{\mu }}_{i}$, ${\hat{\Sigma }}_{i}$, *i* = 1, 2, …, *N*, be the estimated mean and covariance of the data for the *i*^*th*^ segment, respectively. Let the *d* × *p* matrix *B*_1_ be the first *d* rows of *B*. It follows that the mean and variance of $Y_{t}^{s}=B_{1}X_{t}$ on the *i*^*th*^ segment are

(2) $$\begin{array}{r} {\hat{\mu }}_{i}^{s}=B_{1}{\hat{\mu }}_{i}\text{ and }{\hat{\Sigma }}_{i}^{s}=B_{1}{\hat{\Sigma }}_{i}B_{1}^{⊤},i=1,2,\ldots ,N. \end{array}$$

The matrix *B* is then chosen so that the means and covariances vary the least across all epochs. This leads to en estimate *B*_1_ such that *B*_1_*X*_*t*_ is the target stationary source. The objective function is the sum of the Kullback-Leibler (KL) divergences between the $N\left({\hat{\mu }}_{i}^{s},{\hat{\Sigma }}_{i}^{s}\right)i=1,\ldots ,N,$ on each segment and a normal distribution with their grand averages as its parameters, namely $N\left(\bar{\mu^{s}},\bar{\Sigma^{s}}\right)$ where $\bar{\mu^{s}}=1/N\overset{N}{\underset{i=1}{\sum }}{\hat{\mu }}_{i}^{s}$ and $\bar{\Sigma^{s}}=1/N\overset{N}{\underset{i=1}{\sum }}{\hat{\Sigma }}_{i}^{s}$:

(3) $$\begin{array}{l} L\left(B\right)=\overset{N}{\underset{i=1}{\sum }}D_{KL}\left[N\left({\hat{\mu }}_{i}^{s},{\hat{\Sigma }}_{i}^{s}\right)||N\left(\bar{\mu^{s}},\bar{\Sigma^{s}}\right)\right]\\ \;=\overset{N}{\underset{i=1}{\sum }}(-\text{log det}{\hat{\Sigma }}_{i}^{s}+{\left({\hat{\mu }}_{i}^{s}-\bar{\mu^{s}}\right)}^{⊤}\left({\hat{\mu }}_{i}^{s}-\bar{\mu^{s}}\right)). \end{array}$$

The matrix *B* is estimated by minimizing *L*(*B*); see von Bünau et al. (2009) for more details. In the above methodology, the partitioning of the time series into N segments comes with some disadvantages. In addition to the independence assumption, ISSA works under the assumption that nonstationarity in the data is only with respect to the first two moments (mean and variance) which can be restrictive.

### 2.2. Dependent SSA (DSSA)

Here we describe the DSSA approach to finding a stationary subspace process of given multi-dimensional second-order nonstationary processes satisfying Equation (1) using properties from the frequency domain. This method does not require dividing the time series data into several segments and utilizes a test of stationarity for determining whether the estimated source is stationary. This would be useful in not only finding the stationary subspace process (noise reduced signal) but to also ensure there is a statistically significant reduction in the nonstationarity (noise) in the observed EEG signal.

Recall that the discrete Fourier transform (DFT) of any *d*-variate series {*Z*_*t*_}, 1 ≤ *t* ≤ *T*, is given by

(4) $$\begin{array}{r} J_{Z}\left(\omega_{k}\right)=\frac{1}{\sqrt{2\pi T}}\overset{T}{\underset{t=1}{\sum }}Z_{t}\text{exp}\left(-it\omega_{k}\right), \end{array}$$

where $\omega_{k}=\frac{2\pi }{T}k$, *k* = 1, 2, …, *T*. Treating the DFT as a time series indexed by *k*, its lag-*r* autocovariance is the *d* × *d* complex valued matrix given by

(5) $$\begin{array}{r} {\hat{\Gamma }}_{r}^{Z}=\frac{1}{T}\overset{T}{\underset{k=1}{\sum }}J_{Z}\left(\omega_{k}\right)J_{Z}{\left(\omega_{k+r}\right)}^{*}, \end{array}$$

where $J_{Z}{\left(\cdot \right)}^{*}$ denotes the complex conjugate transpose and *r* = 0, 1, …. It is well known (Theorem 4.3.1 of Brillinger, 2001) that for a second-order stationary time series $\left\{Y_{t}^{s}\right\}$, its DFTs are asymptotically uncorrelated when ω_*i*_ ≠ ω_*j*_, i.e.,

(6) $$\begin{array}{r} \text{cov}\left(J_{Y^{s}}\left(\omega_{i}\right),J_{Y^{s}}\left(\omega_{j}\right)\right)=O\left(\frac{1}{T}\right), \end{array}$$

where $J_{Y^{s}}\left(\cdot \right)$ denotes the DFT of of $\left\{Y_{t}^{s}\right\}$. Thus, for a given positive integer *m*, based on the magnitudes of the first few autocovariances of the DFTs of $Y_{t}^{s}=B_{1}X_{t}$, we construct the following objective function as a measure of departure from stationarity:

(7) $$\begin{array}{r} D_{Y}\left(B\right)=\overset{m}{\underset{r=1}{\sum }}||{\hat{\Gamma }}_{r}^{Y^{s}}\left(B\right)|{|}_{F}^{2}, \end{array}$$

where for a matrix *A* ∈ ℝ^*d* × *d*^, $||A|{|}_{F}=\sqrt{\overset{d}{\underset{i,j=1}{\sum }}|a_{ij}^{2}|}$ denotes its Frobenius norm. A solution $\hat{B}$ is obtained by minimizing *D*_*Y*_(*B*) subject to the orthonormality assumption $BB^{⊤}=I_{p}$, see Sundararajan and Pourahmadi (2017) for more details. In section 2.2.5 of the previous work a sequential technique for estimating the unknown dimension *d* of the stationary subspace is described.

The previous work includes theoretical justifications for DSSA to correctly identify the dimension *d* of the stationary source. Also, numerous simulation examples with different types of stationary and nonstationary sources are simulated and the ability of DSSA and ISSA to identify a stationary subspace process $\left\{Y_{t}^{s}\right\}$ and its dimension *d* has been discussed. In these examples, for the nonstationary sources $\left\{Y_{t}^{n}\right\}$ in Equation (1), independent Gaussian components with changing variances and dependent Gaussian components with changing variances are simulated. The dimensions were allowed to vary from 1 to 5. Other simulation examples include stationary vector autoregressive processes (VAR) with no nonstationary sources (*d* = *p*), time-varying vector moving average processes (VMA) with no stationary sources (*d* = 0) and nonstationary unit-root VAR processes with the number of nonstationary sources varying from 1 to *p* − 1.

## 3. Experiment of economic choices: a case study

A total of 181 right-handed students participated in a food snack choice decision experiment conducted in the Texas A&M Human Behavior Laboratory. The sample consisted of about 50% females and 50% males. The subjects were presented with 10 food choice task questions (10 trials). Each choice consisted of two food products, product A and product B. The two products within each alternative had the same features relative to brand, price, packaging and flavor. The only difference between each pair of products was that one of them had fewer calories, making it a healthier choice (original strawberry Jello −70 calories- vs. sugar-free strawberry Jello −10 calories)^1^. Subjects were asked to fast for 3 h prior to the experiment, and received a compensation fee of $20 in exchange for their participation. In order to incentivize and make the food choice task real, one of the 10 tasks was randomly selected to be binding and participants had to eat the food snack before being paid and leaving the laboratory. The displayed picture of each item was the actual photo available for purchase in Walmart's website; however, the participants were not aware that the products were purchased in Walmart.

The experimental design proceeds as follow. At the beginning of the experiment, a blank slide with a fixation point in the middle of the computer screen was presented for 2 s. Then, for each food choice task, the actual product images were presented in the following screen for 8 s. A separate decision slide asked participants which of the two food snacks they prefer to eat. After each decision, an inter-stimulus slide was presented for 0.75 s. The order of the products was randomized across trials in the experimental design; however, all subjects completed the task in the same order.

### 3.1. Data acquisition

The participant was fitted with a proper size EEG headset (B-Alert X10, Advanced Brain Monitoring, Inc.) with 9 electrodes to record brain activity from the pre-frontal (F3, F4, FZ), central (C3, C4, CZ), and parietal (P3, P4, POZ) cortices and a linked mastoid reference. An electrode impedance test was performed to ensure proper conductivity of the electrodes. The impedance level threshold was 20 *k*Ω. An EEG calibration procedure was implemented before the data collection. The EEG calibration incorporated choice tasks (unrelated to the study), psychomotor, and auditory psychomotor vigilance tasks. The EEG data was collected at a sampling rate of 256 Hz. The experiment was presented using the iMotions software platform.

### 3.2. Data analysis

For any given food choice task, say product A vs. product B, we gathered the 9-dimensional EEG signal from the 9 electrodes from the start of the stimuli when the product images are shown to 2.5 seconds after the start. On the digital signal scale, this constitutes 640 observations (2.5 × 256). More precisely, for each subject *j* = 1, 2, …, 181, the data comprises of 640 observations across time.

Given the raw 9-dimensional EEG time series obtained in this case study, we proceed according to the following algorithm to obtain the prediction results:

**Table:** **The Prediction Algorithm**.

Step 1: Filter the raw 9-dimensional signal using a 0.5 Hz high-pass and 45 Hz low-pass filter. Denote the filtered series as {*X*_*t,j*_} where *j* = 1, 2, …, 181 and *t* = 1, 2, …, 640. Step 2: Pre-whiten {*X*_*t,j*_}. For convenience in notation, we denote *X*_*t,j*_ as the band-pass filtered signal that has been pre-whitened. Step 3: Noise reduction: Apply SSA to {*X*_*t,j*_} to obtain {*Y*_*t,j*_} (Section 3.2.1). Step 4: Feature Selection and Prediction Models (Section 3.2.2). Step 5: Assessing Prediction Performance (Section 3.2.3).

In Step 2 we pre-whiten {*X*_*t,j*_} before further analysis by computing the 9 × 9 sample covariance matrix *S*_*j*_ and then transform the data to ${\left(S_{j}\right)}^{-0.5}X_{t,j}$. This standardization reduces the cross-sectional correlation in {*X*_*t,j*_}.

#### 3.2.1. Noise reduction via SSA

It is common to treat data like {*X*_*t,j*_} as a nonstationary time series (Ombao et al., 2005; Park et al., 2014). The words noise and nonstationarity are used interchangeably because in our setup the nonstationary sources contribute to parts of the signal that are unrelated to the food choice task. Hence eliminating nonstationarity reduces noise in the brain signal. As an illustration, we make a plot of the 9-dimensional EEG signal *X*_*t,j*_ (before noise reduction) in Figure 1. In Figure 2, we then plot a 3-dimensional stationary subspace process obtained after application of DSSA. The presence of nonstationarity (noise) in *X*_*t,j*_ was confirmed by carrying out formal tests of stationarity (Jentsch and Subba Rao, 2015). Hence we resort to the SSA technique for removing this nonstationarity from the signal and this is described in this section.

As a pre-processing technique to reduce noise, we apply DSSA and ISSA described in section 2 to obtain a *d* dimensional stationary subspace process where *d* < 9, denoted by {*Y*_*t,j*_}. Since the actual dimension *d* is unknown, we present the results for *d* = 4, 5, 6, 7, 8. We also applied the sequential technique in Sundararajan and Pourahmadi (2017) to detect *d* for each subject and each food choice task. Here we obtained a mode of *d* = 8 as an estimate of the dimension of the stationary subspace.

#### 3.2.2. The prediction models

We discuss three prediction models based on logistic regression with different derived features. The aim of the prediction models discussed below is to fit a model to predict product choice (A or B) based on the input signal. While building prediction models *M*_1_ and *M*_2_, only Step 2 of the algorithm is used, for prediction model *M*_3_ both Steps 2 and 3 are needed. Note that model *M*_2_ assumes that {*X*_*t,j*_} is stationary whereas model *M*_3_ assumes that {*X*_*t,j*_} is nonstationary and applies SSA before extracting features and estimating the prediction model.

##### 3.2.2.1. Model M_1_

A standard model similar to Telpaz et al. (2015) is based on the importance of the pre-frontal EEG channels in explaining choice behavior in individuals. Following their aggregation technique to reduce the noise when computing preference scores for products, we take average of the signals from the 3 pre-frontal channels (F3, F4, FZ) over the 2.5 s. The signal here is a 3-dimensional band-pass filtered signal that was pre-whitened. The average is taken per subject per food choice question (say product A vs. product B). For subject *j*, *j* = 1, 2, …, 181 , this average denoted by the scalar ${\bar{X}}_{j}$ is used as a feature in the following logistic regression model:

(8) $$\begin{array}{r} P(c_{j,AB}=1|{\bar{X}}_{j})=\frac{exp\left(a_{0}{\bar{X}}_{j}\right)}{1+exp\left(a_{0}{\bar{X}}_{j}\right)}, \end{array}$$

for *j* = 1, 2, …, 181. In the model above we have denoted 1 for product A and 0 for product B and the model predicts the class (0 or 1) based on the derived feature ${\bar{X}}_{j}$.

##### 3.2.2.2. Model M_2_

In this approach, to distinguish between the two classes denoted as 1 for product A and 0 for product B, we take {*X*_*t,j*_} the pre-whitened 9-dimensional band-pass filtered signal. We then focus on the covariance structure of *X*_*t,j*_ for each of the two classes (0 and 1). The aim is to derive features that bring out the differences between the two classes based on the covariance structure of the signal. This is achieved by computing the average spectral density matrices for the two classes over the Fourier frequencies:

(9) $$\begin{array}{r} \bar{g^{i}}\left(\omega_{k}\right)=\frac{1}{n_{i}}\underset{j\in \text{Class i}}{\sum }g_{j}\left(\omega_{k}\right),i=0,1, \end{array}$$

where *g*_*j*_(ω_*k*_) is the estimated 9 × 9 spectral matrix for subject *j* using observations {*X*_*t,j*_}, *n*_*i*_ for *i* = 0, 1 is the number of subjects in the two classes and $\omega_{k}=\frac{2\pi k}{640}$, *k* = 1, 2, …, 640, are the fundamental Fourier frequencies. The spectral matrix was estimated using a Daniell kernel with smoothing window length 25 (approximately $\sqrt{640}$); see Example 10.4.1 in Brockwell and Davis (1991).

In order to train the classifier, for every subject *j* ∈ {1, 2, …, 181}, a distance vector *p*_*j,AB*_ = (*p*_0,*j,AB*_, *p*_1,*j,AB*_) is computed where

$$\begin{array}{l} p_{i,j,AB}=\frac{1}{640}\overset{640}{\underset{k=1}{\sum }}||g_{j}\left(\omega_{k}\right)-\bar{g^{i}}\left(\omega_{k}\right)|{|}_{F}^{2}i=0,1. \end{array}$$

and ||·||_*F*_ is the Frobenius norm of a matrix. It measures the distance to the center of each of the two classes and serves as our two-dimensional feature vector used in constructing the following logistic regression model (prediction model):

(10) $$\begin{array}{r} P(c_{j,AB}=1|p_{j,AB})=\frac{exp\left(\alpha_{0}p_{0,j,AB}+\alpha_{1}p_{1,j,AB}\right)}{1+exp\left(\alpha_{0}p_{0,j,AB}+\alpha_{1}p_{1,j,AB}\right)}, \end{array}$$

for *j* = 1, 2, …, 181 and *c*_*j, AB*_ is the class indicator (1 for product A or 0 for product B) for subject *j*.

##### 3.2.2.3. Model M_3_

Here we apply Step 2 on the raw 9-dimensional EEG signal to obtain {*X*_*t,j*_}. Then we obtain on the *d*-variate stationary subspace processes, {*Y*_*t,j*_}, using DSSA/ISSA (Step 3). Similar to the approach in model *M*_2_, we aim to capture the differences between the two classes based on the covariance structure of the signal. Unlike model *M*_2_, we apply DSSA and ISSA described in section 2 to obtain a *d*-dimensional stationary subspace process where *d* < 9, denoted by {*Y*_*t,j*_}. Features to be fed into the prediction model will be based on {*Y*_*t,j*_} as opposed to model *M*_2_ wherein {*X*_*t,j*_} was used. Then, proceeding as in model *M*_2_, we compute the average spectral density matrices for the two classes over the Fourier frequencies:

(11) $$\begin{array}{r} \bar{f^{i}}\left(\omega_{k}\right)=\frac{1}{n_{i}}\underset{j\in \text{Class i}}{\sum }f_{j}\left(\omega_{k}\right),i=0,1, \end{array}$$

where *f*_*j*_(ω_*k*_) is the estimated *d* × *d* spectral matrix for subject *j* using observations {*Y*_*t,j*_}, *n*_*i*_ for *i* = 0, 1 is the number of subjects in the two classes and $\omega_{k}=\frac{2\pi k}{640}$, *k* = 1, 2, …, 640 are the fundamental Fourier frequencies. The spectral matrix was estimated using a Daniell kernel with smoothing window length 25 (approximately $\sqrt{640}$).

In order to train the classifier, for every subject *j* ∈ {1, 2, …, 181}, a distance vector *d*_*j,AB*_ = (*d*_0,*j,AB*_, *d*_1,*j,AB*_) is computed where

$$\begin{array}{l} d_{i,j,AB}=\frac{1}{640}\overset{640}{\underset{k=1}{\sum }}||f_{j}\left(\omega_{k}\right)-\bar{f^{i}}\left(\omega_{k}\right)|{|}_{F}^{2}i=0,1. \end{array}$$

and ||·||_*F*_ is the Frobenius norm of a matrix. It measures the distance to the center of each of the two classes and serves as our two-dimensional feature vector used in constructing the following logistic regression model (prediction model):

(12) $$\begin{array}{r} P(c_{j,AB}=1|d_{j,AB})=\frac{exp\left(\beta_{0}d_{0,j,AB}+\beta_{1}d_{1,j,AB}\right)}{1+exp\left(\beta_{0}d_{0,j,AB}+\beta_{1}d_{1,j,AB}\right)}, \end{array}$$

for *j* = 1, 2, …, 181 and *c*_*j, AB*_ is the class indicator (1 for product A or 0 for product B) for subject *j*.

#### 3.2.3. Prediction performance

We asses the performance by computing the overall prediction accuracy and the average sensitivity and specificity. Using the confusion matrix given in Table 1, we compute two prediction accuracy measures given by

(13) $$\begin{array}{r} A_{1}=\frac{C_{A}+C_{B}}{T_{A}+T_{B}},A_{2}=\frac{\frac{C_{A}}{T_{A}}+\frac{C_{B}}{T_{B}}}{2}, \end{array}$$

where *A*_1_ is the overall prediction accuracy of the model and *A*_2_, in a binary classification context, is the average of sensitivity (true positive rate) and specificity (true negative rate) of the prediction models. Finally, we present an estimate of the AUC: area under the ROC curve (LeDell et al., 2015) for the 10 food choice questions for each of the 3 models and this measure is denoted as *A*_3_. The ROC curve plots the true positive rate against the false positive rate and is a useful measure of model performance. The area under the ROC curve (known as AUC) varies between 0 and 100% with a value of 50% as baseline (uninformative classifier).

**Table 1:** Confusion matrix.

		**Prediction**		
		**Product A**	**Product B**	**Total**
Actual	Product A	*C*_*A*_	*I*_*B*_	*T*_*A*_
	Product B	*I*_*A*_	*C*_*B*_	*T*_*B*_

In Table 2, we shuffle the class labels randomly and fit the prediction models and assess the performance measures. The shuffling of labels is done 500 times, each time fitting the prediction models, and the average performance measure over the 500 runs across the 10 food choice questions is presented. This enables us to identify a baseline for the 3 performance measures (70% for performance measure *A*_1_ and 50% for performance measures *A*_2_ and *A*_3_).

**Table 2:** Prediction performance of the 3 models with shuffled labels: the average of the 3 performance measures *A*_1_, *A*_2_, and *A*_3_ (AUC) taken across the 10 food choice questions for the three competing models *M*_1_, *M*_2_, and *M*_3_.

**Model**	**Overall accuracy - *A*_1_**	**Avg. of sensitivity and specificity - *A*_2_**	**AUC - *A*_3_**
*M*_1_	69.54	48.97	53.26
*M*_2_	69.05	51.81	53.25
*M*_3_ - DSSA	69.12	51.34	54.20
*M*_3_ - ISSA	69.51	51	52.08

*For model M_3_ the choice of d is taken as 8*.

These accuracy rates are computed using a 10-fold cross-validation technique where the data is randomly divided into 10 nearly equal parts. Each part is removed, in turn, while the remaining data are used to fit the prediction models *M*_1_, *M*_2_, *M*_3_ and the predictions are carried out for the left out part. More precisely, the computed accuracy rates are the out-of sample estimates wherein for any given pair of products A and B, the prediction model is fit based on roughly 90% of the subjects and the predictions are carried out for the remaining subjects.

**Figure 3:**
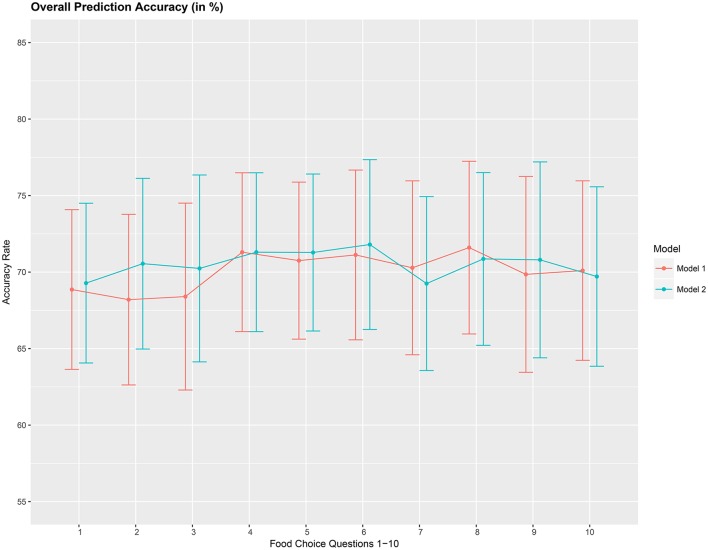
Overall prediction accuracy rate (*A*_1_), in %, based on a 10-fold cross-validation for the 10 food choice tasks for the two mdoels *M*_1_ and *M*_2_. Approximate 95% confidence intervals included for each accuracy estimate.

**Figure 4:**
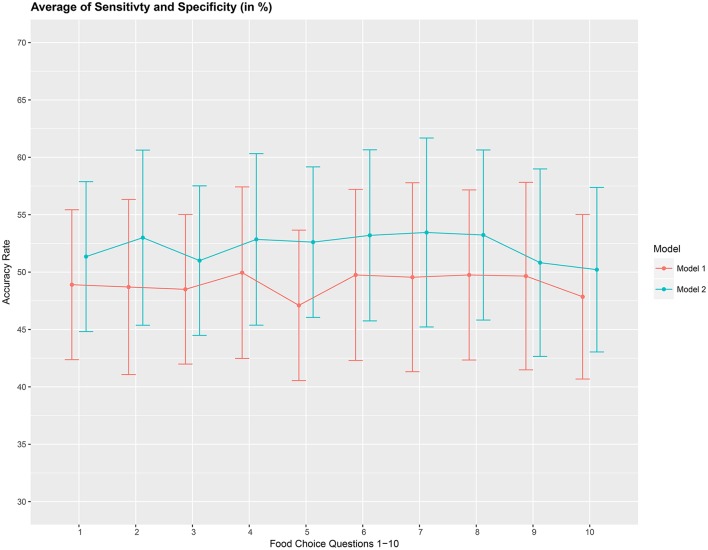
Prediction accuracy rate as average of sensitivity and specificity (*A*_2_), in %, based on a 10-fold cross-validation for the 10 food choice questions for the two models *M*_1_ and *M*_2_. Approximate 95% confidence intervals included for each accuracy estimate.

The overall accuracy rate (*A*_1_) for models *M*_1_ and *M*_2_ computed and plotted in Figure 3 shows that it varies between 69 and 72% for both models. Next, we look at the performance measure *A*_2_ as an average of the sensitivity and specificity of models *M*_1_ and *M*_2_. We notice from Figure 4 that both methods perform poorly with accuracy rates around 50%. Note that as opposed to averaging over the signal across the 3 channels in model (Equation 8), we also assessed the performance of the logistic regression models fitted individually with each of the pre-frontal channels. We obtained rates (not presented here) similar to that seen in Figures 3, 4 in terms of overall prediction accuracy and average of sensitivity and specificity.

**Figure 5:**
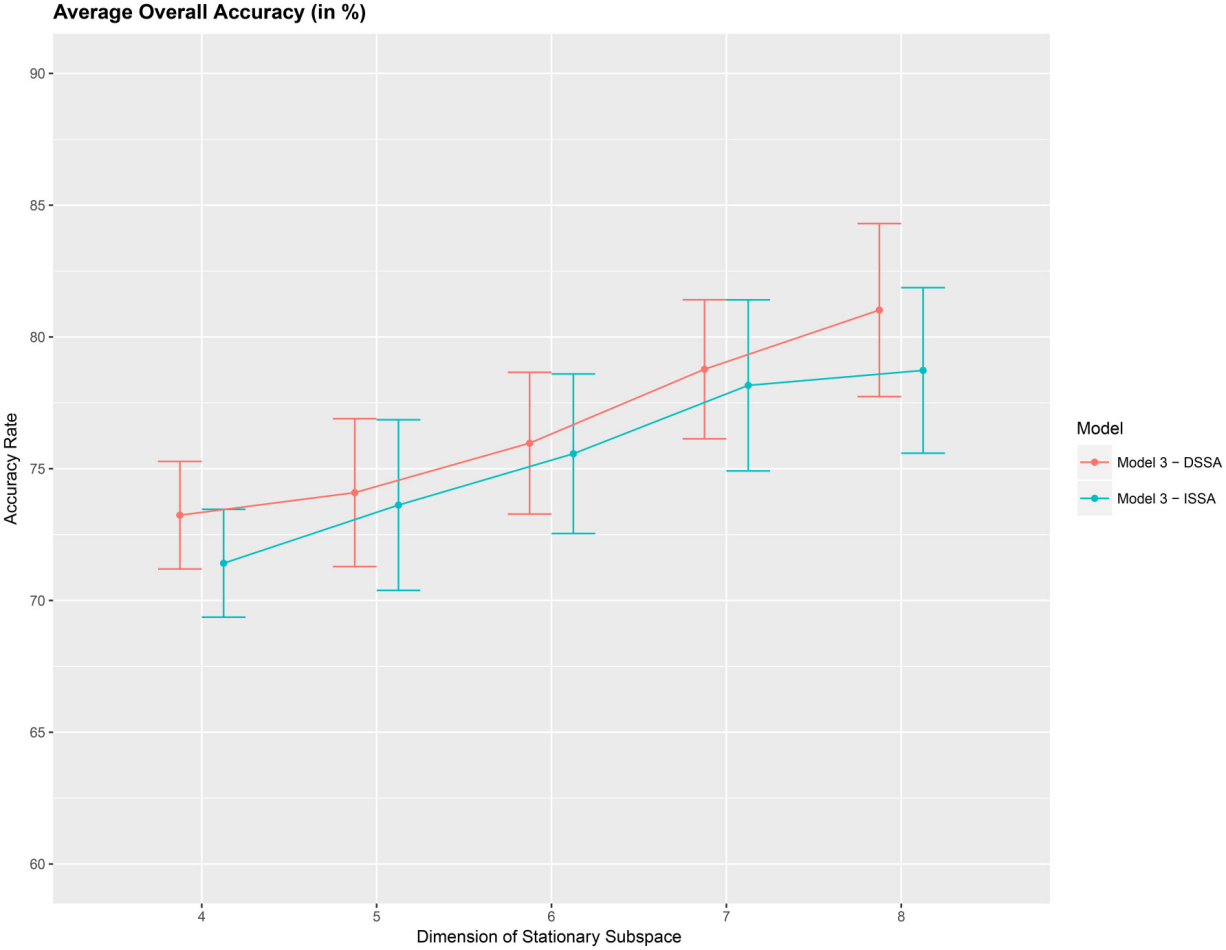
Average 10-fold cross-validation overall accuracy rate (in %) for the 10 food choice questions (y-axis) vs. dimension of the stationary subspace (x-axis). Approximate 95% confidence intervals included for each accuracy estimate.

Next, we study the overall prediction accuracy (*A*_1_) after applying the pre-processing techniques DSSA and ISSA and removing the nonstationarity (noise) in the EEG signal, and fitting the prediction model (Equation 12 of model *M*_3_). Since the actual dimension of the stationary subspace is unknown, in Table 3 we present the results for dimensions *d* = 4, 5, 6, 7, 8, which show that DSSA performs better than ISSA in most cases. The average overall accuracy rate based on the 10 food choice tasks for each value of *d* is given in Figure 5. It is seen that the 10-fold cross-validation accuracy rate is around 80% for each of the 10 tasks when the dimension *d* = 8. This rate is roughly 10% more than the accuracy rate from Figure 3 wherein no SSA-type pre-processing technique is applied. We also notice that as the dimension of the stationary subspace *d* increases, the accuracy rate also increases. This phenomenon was also observed in von Bünau et al. (2010) and confirms the improvements in prediction accuracy when there are fewer nonstationary sources (noise) in model (Equation 1). The DSSA/ISSA turns out to be a very useful tool for reducing the noise (nonstationarity) in the EEG signal.

**Table 3:** Ten-fold cross-validation overall prediction accuracy (in %) for the 10 questions Q1–Q10 corresponding to *d* = 4, 5, 6, 7, 8 for DSSA and ISSA (model *M*_3_).

***d***		**Q1**	**Q2**	**Q3**	**Q4**	**Q5**	**Q6**	**Q7**	**Q8**	**Q9**	**Q10**
4	DSSA	**72.80**	**73.65**	74.22	71.53	**74.00**	73.83	**73.48**	**71.38**	**74.50**	72.97
	ISSA	69.60	71.20	75.10	71.40	66.50	76.20	70.18	68.45	72.37	73.13
5	DSSA	73.50	73.21	**75.76**	**73.00**	68.30	**77.85**	73.55	72.40	**74.60**	**78.75**
	ISSA	74.00	75.48	71.80	71.80	75.10	75.10	75.20	74.62	72.40	70.70
6	DSSA	**74.65**	75.00	**78.46**	72.85	77.35	**75.10**	75.70	74.52	**77.45**	**78.61**
	ISSA	71.94	77.30	74.00	79.62	78.50	73.50	75.70	76.80	74.00	74.25
7	DSSA	76.12	**80.27**	**78.56**	79.15	79.20	76.15	79.65	80.11	**79.12**	**80.50**
	ISSA	75.70	74.00	76.20	81.80	80.12	79.80	78.50	80.80	77.90	76.80
8	DSSA	**78.98**	**81.35**	**81.52**	**84.12**	**80.24**	79.11	**82.30**	80.58	80.13	**81.94**
	ISSA	76.90	75.10	80.16	79.60	75.10	79.00	79.00	84.75	82.00	75.68

*Significant results (instances of at least a 1% improvement in DSSA) are highlighted in bold*.

**Figure 6:**
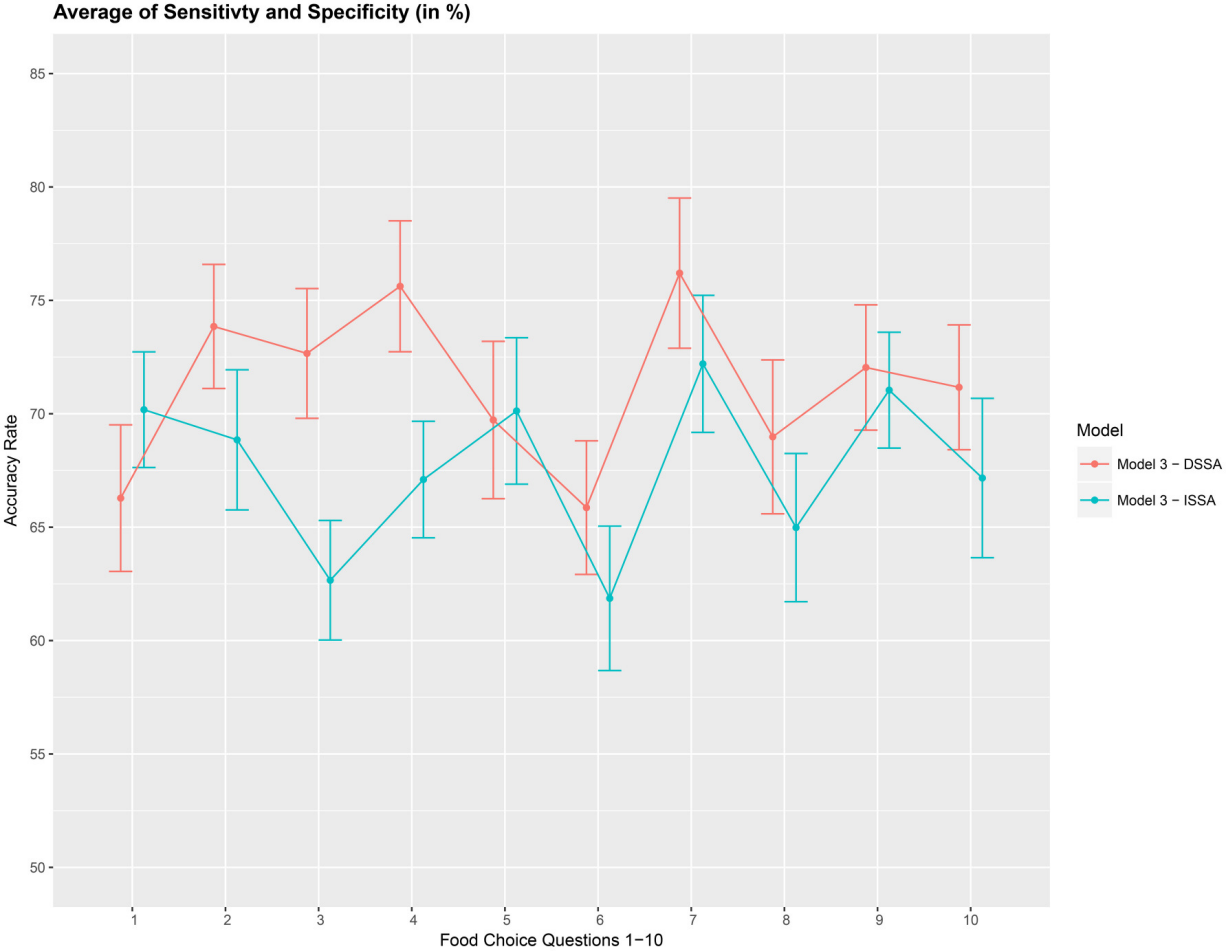
Prediction accuracy rate: average of sensitivity and specificity (*A*_2_), in %, based on a 10-fold cross-validation for the 10 food choice questions. Model *M*_3_ was used with *d* = 8. Approximate 95% confidence intervals included for each accuracy estimate.

We then asses the performance measure *A*_2_ which is an average of the sensitivity and specificity for model *M*_3_. We set the dimension of the stationary subspace at *d* = 8. Figure 6 shows that DSSA performs slightly better than ISSA in most cases. More importantly, we note that in comparison to Figure 4, DSSA has roughly a 20% increase in the performance measure *A*_2_.

**Figure 7:**
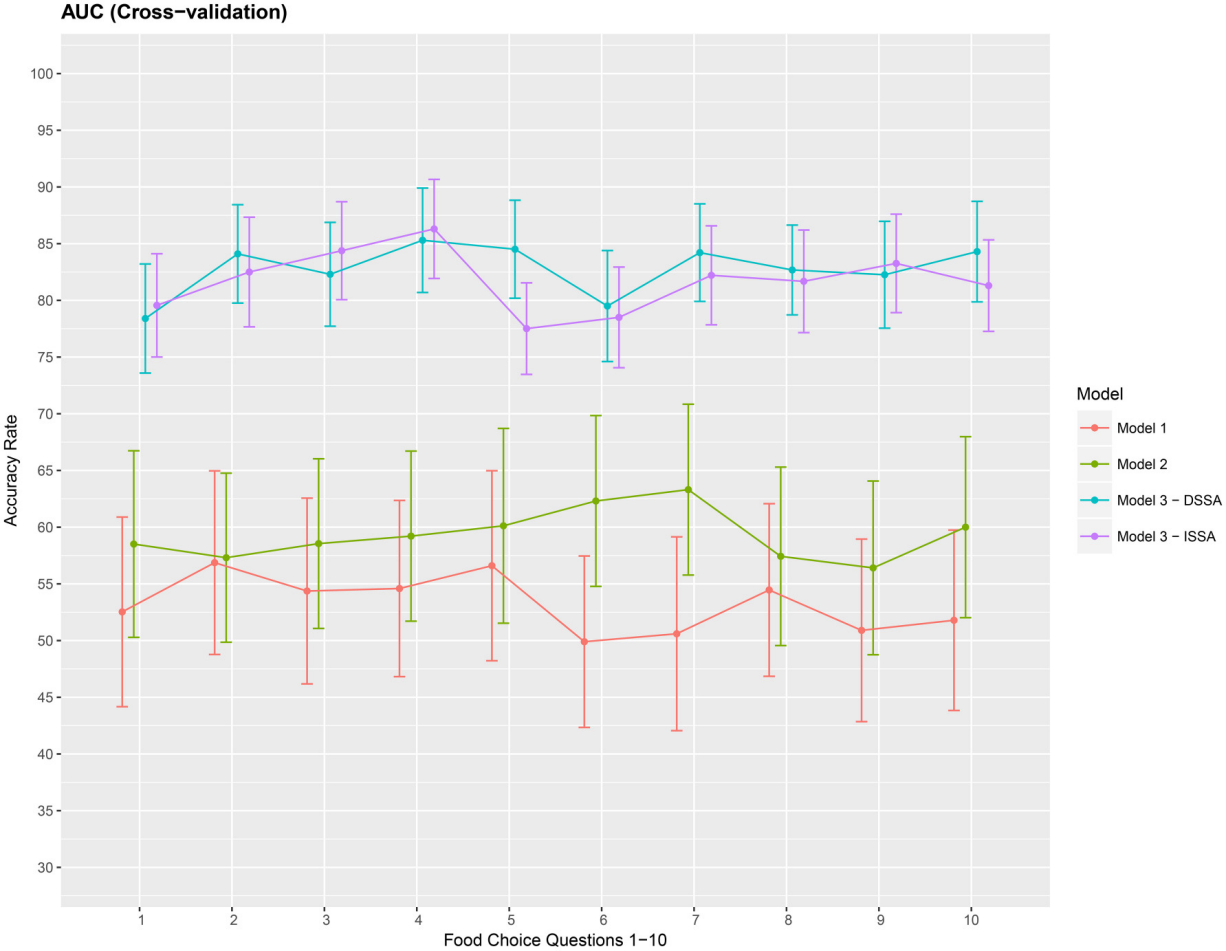
Cross-validation estimate of the AUC in % (Area under the ROC curve) for the 3 models *M*_1_ *M*_2_ and *M*_3_. Approximate 95% confidence intervals included for each accuracy estimate. For model *M*_3_ we take *d* = 8.

Finally, we present a cross-validation estimate of the AUC for the 3 competing models in Figure 7. We again notice roughly a 20% increase when using DSSA/ISSA (Model *M*_3_) as a noise reduction technique before constructing the prediction model.

The average of the 3 performance measures *A*_1_, *A*_2_, and *A*_3_(AUC) taken across the 10 food choice questions for the three competing models *M*_1_, *M*_2_, and *M*_3_ is reported in Table 4. We note that for models *M*_1_ and *M*_2_ the overall prediction accuracy (*A*_1_) is roughly 70% which is treated as a baseline for this measure. However, the performance measures *A*_2_ and *A*_3_ (AUC) are only around 50% which suggests a poor performance. In contrast for model *M*_3_, overall accuracy rate increased by roughly 10%, the measure *A*_2_ is higher by around 20% and measure *A*_3_ (AUC) is significantly higher (increase of roughly 30%) than models *M*_1_ and *M*_2_.

**Table 4:** The average of the 3 performance measures *A*_1_, *A*_2_, and *A*_3_ (AUC) taken across the 10 food choice questions for the three competing models *M*_1_, *M*_2_, and *M*_3_.

**Model**	**Overall accuracy - *A*_1_**	**Avg. of sensitivity and specificity - *A*_2_**	**AUC - *A*_3_**
*M*_1_	70.04	48.97	53.26
*M*_2_	70.52	52.17	59.31
*M*_3_ - DSSA	81.02	71.23	82.75
*M*_3_ - ISSA	78.73	67.61	81.72

*For model M_3_ the choice of d is taken as 8*.

## 4. Concluding remarks

EEG records the electrical activity of the brain directly in the scalp. EEG signals have high temporal resolution, thus providing rich time series data of brain activity. We concentrate on EEG because it is a less expensive method to obtain brain data, making it more accessible. Brain data, however, is inherently noisy, because it captures the brain activity for the stimuli, along with other activity unrelated to the task of the experiment. In order to filter out the noise, neuroeconomic experiments typically aggregate data from hundreds of trials from each participant. In addition to potential fatigue effects, we point out a tradeoff between the basic experimental economics principle of simplicity with the neuroeconomic need for a large number of trials to reduce brain signal noise. We apply a new statistical technique to a food choice task and show its potential for reducing the noise and hence the number of trials needed for EEG experiments. Based on the results presented in section 3.2.3, we notice that the overall accuracy rate is around 80% for each of the 10 trials when noise reduction is carried out through SSA (model *M*_3_). More importantly, the overall prediction accuracy from a single trial increased by around 10%, the average of sensitivity and specificity increased by around 20% and the AUC increased by roughly 30% when the DSSA/ISSA technique was used to reduce signal noise.

The improvement in the prediction results in this case study by implementing noise reduction via DSSA/ISSA indicates the dynamic behavior of the brain processes. This time-varying behavior leads to nonstationarity in the observed multi-dimensional EEG signal. This phenomenon has been observed and studied in other works (Ombao et al., 2005; Demiralp et al., 2007; von Bünau et al., 2009; Sundararajan and Pourahmadi, 2017, to name a few) wherein the observed EEG signal is treated as a multi-dimensional nonstationary time series. Hence removing nonstationarity from the EEG signal is seen to improve prediction performance in the above cited works and also in the current case study. The improvement in the prediction performance of EEG brain data shown in this case study is encouraging. Future work can build upon our procedure to develop other post-processing techniques to further refine neurophysiological predictors of choice behavior.

## Ethics statement

The Study was approved by the IRB protocol number IRB2016-0059D.

## Author contributions

The research problem and data came from the second author (Human Behavior Laboratory at Texas A&M). The methodology and computations were performed by the first and third authors. Contributions toward presentation of the results and connecting the research problem to the methodology came from all three authors. All authors have made direct and intellectual contribution to the work, and approved it for publication.

### Conflict of interest statement

The authors declare that the research was conducted in the absence of any commercial or financial relationships that could be construed as a potential conflict of interest.

## Footnotes

^1^The product list and amount of calories is listed in section A.1 in Appendix. The focus of this paper is reducing EEG noise to improve the prediction of which of the two food snacks participants would choose, irrespective of the product's identity.

## A. Appendix

### A.1. List of food snack products used in the experiment

#### A.1.1. Experimental instructions

The Food Choice Task will proceed as follows:

1. This stage consists of 10 choice situations.
2. In each trial, you will be presented with two food products.
3. You need to choose which of the two products you would prefer to eat.
4. Your decisions are real. At the conclusion of the experiment, one decision will be randomly selected to be binding.
5. You will receive one single unit of the food product you chose and will have to eat it at the end of today's session.

**Table A1:** Food snack choices.

**Choice**	**Product A**	**Product B**
1	Low calorie Jell-O gelatin (10 cal.)	Original Jell-O gelatin (70 cal.)
2	Oven baked Lays potato chips (120 cal.)	Classic Lays potato chips (160 cal.)
3	No sugar added Dole peaches (25 cal.)	Original Dole peaches (70 cal.)
4	Light Yoplait yogurt (90 cal.)	Original Yoplait yogurt (150 cal.)
5	Fat free Pringles potato chips (70 cal.)	Original Pringles potato chips (150 cal.)
6	Sugar free Snack Pack pudding (70 cal.)	Original Snack Pack pudding (110 cal.)
7	Reduced fat Sargento string cheese (50 cal.)	Original Sargento string cheese (80 cal.)
8	Non-fat Oikos Greek yogurt (120 cal.)	Traditional Oikos Greek yogurt (150 cal.)
9	Reduced fat Cheez-It baked crackers (130 cal.)	Original Cheez-It baked crackers (150 cal.)
10	Diet Lipton green tea (0 cal.)	Traditional Lipton green tea (100 cal.)

*The pictures of all products were taken from the WalMart's website where they were purchased*.
